# Oral anticancer agent medication adherence by outpatients

**DOI:** 10.3892/ol.2014.2480

**Published:** 2014-08-26

**Authors:** MICHIO KIMURA, EISEKI USAMI, MINA IWAI, TOSHIYA NAKAO, TOMOAKI YOSHIMURA, HIROMI MORI, TADASHI SUGIYAMA, HITOMI TERAMACHI

**Affiliations:** 1 Department of Pharmacy, Ogaki Municipal Hospital, Ogaki, Gifu, Japan; 2 Laboratory of Clinical Pharmacy Practice and Social Science, Gifu Pharmaceutical University, Gifu, Japan; 3 Laboratory of Clinical Pharmacy, Gifu Pharmaceutical University, Gifu, Japan

**Keywords:** adherence, compliance, oral anticancer agent, customer satisfaction

## Abstract

In the present study, medication adherence and factors affecting adherence were examined in patients taking oral anticancer agents. In June 2013, 172 outpatients who had been prescribed oral anticancer agents by Ogaki Municipal Hospital (Ogaki, Gifu, Japan) completed a questionnaire survey, with answers rated on a five-point Likert scale. The factors that affect medication adherence were evaluated using a customer satisfaction (CS) analysis. For patients with good and insufficient adherence to medication, the median ages were 66 years (range, 21–85 years) and 73 years (range, 30–90 years), respectively (P=0.0004), while the median dosing time was 131 days (range, 3–3,585 days) and 219 days (24–3,465 days), respectively (P=0.0447). In 36.0% (62 out of 172) of the cases, there was insufficient medication adherence; 64.5% of those cases (40 out of 62) showed good medication compliance (4–5 point rating score). However, these patients did not fully understand the effects or side-effects of the drugs, giving a score of three points or less. The percentage of patients with good medication compliance was 87.2% (150 out of 172). Through the CS analysis, three items, the interest in the drug, the desire to consult about the drug and the condition of the patient, were extracted as items for improvement. Overall, the medication compliance of the patients taking the oral anticancer agents was good, but the medication adherence was insufficient. To improve medication adherence, a better understanding of the effectiveness and necessity of drugs and their side-effects is required. In addition, the interest of patients in their medication should be encouraged and intervention should be tailored to the condition of the patient. These steps should lead to improved medication adherence.

## Introduction

Medication adherence is often defined as follows: Subsequent to sufficient explanation of the effects and side-effects of medication, the patient agrees to be treated with the medication, understands the significance of the medication and continues to take the medication voluntarily (1). By contrast, the conventionally used definition of medication compliance refers to the patient taking medication in the amount and at the times directed by pharmacists and physicians. Cancer treatment through oral anticancer agents has the advantage of ease of delivery, but since medication management is left to the family or patient, medication adherence can be a problem. It is important that the patient understands the effects of the prescribed drugs, the side-effects and the methods to assuage these, and that they also understand that medication adherence can lead to effective treatment, safety and continuity in cancer chemotherapy. A lack of medication adherence can decrease treatment efficiency, change the seriousness of the side-effects and increase the number of hospitalizations and doctor visits, all of which can lead to higher medical costs (2,3). Therefore, the improvement of medication adherence is an important component in cancer treatment.

Patients who are diagnosed with cancer are generally observed as having strong reasons for adhering to treatment, as a lack of adherence could lead to serious side-effects, cancer relapse or mortality. Despite the serious effects of non-adherence, previous studies have indicated that the medication adherence rate in cancer patients is not 100% (4–8).

Research into medication adherence in patients taking oral anticancer agents has generally focused on a specific disease, such as chronic myeloid leukemia (CML) or breast cancer, and the disease-specific medicine (4–8). At present, neither the adherence to tegafur/gimeracil/oteracil potassium (S-1) and multiple tyrosine kinase inhibitors nor the level of consciousness behind non-adherence or non-compliance has been examined. Therefore, a questionnaire survey was conducted to evaluate the factors that affect medication adherence in patients taking oral anticancer agents. The results of the survey were explored using a customer satisfaction (CS) analysis.

## Materials and methods

### Participants

The participants in the present study were outpatients undergoing treatment with oral anticancer agents at Ogaki Municipal Hospital (Ogaki, Gifu, Japan) in June 2013. A self-report questionnaire survey was administered to these patients, and those who had difficulty reading or writing were assisted. The questionnaires were distributed to 182 individuals. The return rate for the questionnaires was 94.5% (172 out of 182).

The present study was approved by the Institutional Review Board of Ogaki Municipal Hospital and was explained in a handout distributed to participants prior to obtaining their informed consent.

### Measures

#### Questionnaire survey items

The questionnaire items are shown in Table I. The survey items included: Medication adherence (six items total), with one item each for the dosing method, effect of drugs, side-effects, understanding of the treatment method, treatment policy and compliance; personality (three items); and factors potentially affecting adherence, namely, the living environment (one item), awareness of medication dosage (three items), knowledge of the drug (six items), daily schedule (two items), understanding of the disease (one item), sense of trust (two items), expectations and attitude (two items) and condition (one item). The participants were instructed to include all currently prescribed medications in one of the dosing method questions.

The survey items were rated on a five-point Likert scale, with 5 being ‘Yes’, 4 being ‘I think so’, 3 being ‘I cannot say either way’, 2 being ‘I do not think so’ and 1 being ‘No’. Items 9–11 were reverse-scored.

Using the scores on the medication adherence scale of the questionnaire, the participants were divided into two groups: The ‘good medication adherence’ group, consisting of those who adhered to medication regimens, with a score ≥4 on all items; and the ‘insufficient medication’ group, which consisted of those who did not adhere, classified as participants who scored ≤3 on all items. The scores across the two groups were compared for each variable. All data, including the evaluation score value, the number of drugs taken and the dosing time, are presented as the median value within the range.

#### Evaluation of medication adherence by CS analysis

CS object variable analysis was performed with the lowest point for each item in medication adherence in order to investigate adherence factors, and the questions associated with the factors that potentially affected medication adherence were analyzed to determine an explanatory variable. For the questions associated with the factors that potentially affected medication adherence, CS analysis was performed with the items that possessed significant differences between the good and insufficient groups.

The CS analysis graph was plotted on two-dimensional coordinates. The average deviations of the scores for each item on the questionnaire were plotted on the vertical axis of the CS analysis graph, while the association value (correlation coefficient deviation value) between adherence factors and the individual evaluation was plotted on the horizontal axis. In the CS analysis graph, factors with a high score and a high degree of influence on adherence are plotted in the first quadrant, termed the emphasis maintenance field. Factors with a high score and low degree of influence on adherence are plotted in the second quadrant, termed the maintenance field. Factors with a low score and low degree of influence on adherence are plotted in the third quadrant, termed the improvement field, and factors with a low score and high degree of influence on adherence are plotted in the fourth quadrant, termed the priority improvement field. The CS analysis improvement degree for each question was calculated. This degree is an index indicating the magnitude of the effect a factor has on adherence. If the CS analysis improvement degree is positive, improvement is required in the items with a score of five points or greater. Conversely, a negative CS analysis improvement degree would indicate that improvement is not necessary.

#### Statistical analysis

The Mann-Whitney U test was used to compare the two groups. In all statistical tests, P<0.05 was considered to indicate a statistically significant difference. For the CS analysis, the statistical software, EXCEL^®^ Quality Management (Esumi Co., Ltd., Tokyo, Japan), was used.

## Results

### Drugs

The drugs represented in the current study and the number of patients taking those drugs were S-1 (n=83), capecitabine (n=26), molecularly-targeted drugs, such as sorafenib (n=45), tegafur-uracil combination (n=10) and other drugs, including cyclophosphamide, mercaptopurine and hydroxycarbamide (n=10).

### Evaluation of medication adherence

**Figure 1:**
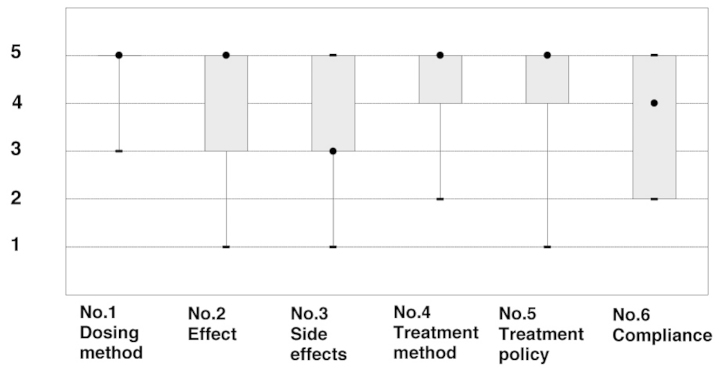
Evaluation scores of insufficient medication adherence patients. The black circle (●) in the boxes signifies a median value, the line under the box represents the first quartile, and the line above the box represents the third quartile. The lines extending above and below the box indicate the maximum and minimum values.

Good medication adherence was found for 64.0% of the patients (110 out of 172). The scores of the patients with insufficient adherence to medication are shown in Fig. 1. The scores [median (range)] were 5.0 (3.0–5.0) for the dosing method, 5.0 (1.0–5.0) for the effect of the drug, 3.0 (1.0–5.0) for the side-effects, 5.0 (2.0–5.0) for the understanding of the treatment method, 5.0 (1.0–5.0) for the treatment policy and 4.0 (2.0–5.0) for compliance.

In 36.0% (62 out of 172) of the cases, there was insufficient medication adherence. However, 64.5% of those cases (40 out of 62) had good medication compliance, with a score of 4–5 points. It is likely that these patients did not fully understand the effects of the drugs or side-effects, giving a score of three points or less on these items. However, the percentage of patients with good medication compliance was 87.2% (150 out of 172).

### Evaluation of patient demographic factors that affect medication adherence

The patient demographics that potentially affected medication adherence are shown in Table II. Patients with good and insufficient adherence to medication had a median age of 66 years (range, 21–85 years) and 73 years (range, 30–90 years), respectively (P=0.0004). The median number of drugs taken was four for each group (good adherence range, 1–10 drugs; insufficient adherence range, 1–14 drugs; P=0.0401) and the length of time on the medication was 131 days (range, 3–3,585 days) in the good adherence to medication group and 219 days (24–3,465 days) (P=0.0447) in the insufficient adherence group.

### Evaluation of factors that affect medication adherence

The scores associated with factors that affected medication adherence are shown in Table III. For patients with good or insufficient adherence to medication, there were significant differences (P<0.05) in nine items on the following subscales: Awareness of dosing (one out of three items), awareness about the drug (three out of six items), understanding the disease (one out of one item), sense of trust (two out of two items), expectations and attitudes (one out of two items) and the condition of the patient (one out of one item).

### CS analysis on the improvement of medication adherence

**Figure 2:**
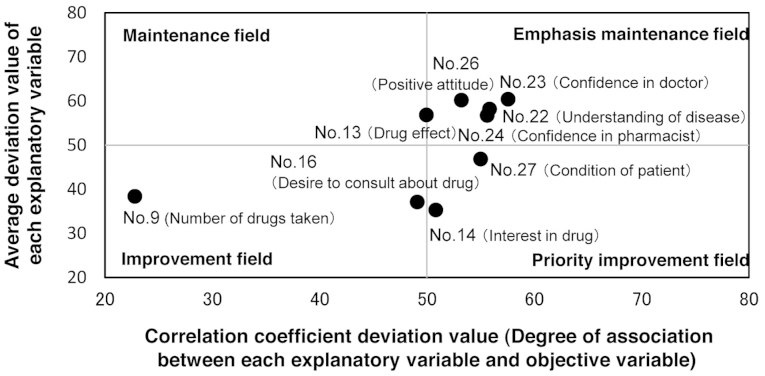
Customer satisfaction (CS) analysis graph. The CS analysis graph was plotted on two-dimensional coordinates. The average deviation value of the scores for each question item in the questionnaire are plotted on the vertical axis of the CS analysis graph and the association value (correlation coefficient deviation value) between adherence factors and the individual evaluation are shown on the horizontal axis of the CS analysis graph.

The CS analysis graph is shown in Fig. 2 and the data obtained from CS analysis are shown in Table IV. From the CS analysis, three items, namely interest in the drug, desire to consult about the drug and condition of the patient, were found to be areas requiring improvement (degree of improvement; 11.00, 8.50, and 5.77, respectively).

## Discussion

Numerous studies have examined medication adherence in patients taking oral anticancer agents for CML and breast cancer (5,7–13). These studies have reported considerably different medication adherence rates; for example, the adherence rate of patients with CML was 14.2% in a study by Noens *et al* (7), but 98% in a study by Marin *et al* (5). Thus, it may be that differences in medication adherence rates depend largely on the survey method. In the present study, the percentage of patients with good medication adherence was not high (64.0%), however, the percentage of patients with good medication compliance was 87.2%. As numerous patients took the medication as directed, it can be concluded that the medication compliance of patients taking oral anticancer agents was good, but that the medication adherence, according to the definition of the present study, was insufficient.

Medication non-adherence in cancer chemotherapy can lead to an increase in the seriousness of side-effects, a deterioration in general health and a worse prognosis. Therefore, it is important to learn the causes of non-adherence to improve overall adherence.

The present study found that medication adherence decreased with age. Hasegawa *et al* (14) and Tsuboi *et al* (15) reported that medication adherence is higher in elderly patients compared with young patients. These differences may be accounted for by differences in the participants, but since this is not the only possibility, differences in participants should be a topic for future investigations. In addition, the patients with a longer course of medication were more likely to be in the insufficient medication adherence category. Ziller *et al* (16) reported that when patients received supplementary information, medication adherence was good after 12 months. Therefore, in cases with a long course of medication, patient guidance and information is important.

With regard to the factors affecting medication adherence, nine items that had a significant effect on adherence were observed in the present study: The number of drugs taken, the effect of the drug, researching the medication, consulting with the doctor or pharmacist about the drug, the understanding of the disease, confidence in the doctor, confidence in the pharmacist, a positive attitude and the condition of the patient. Russmann *et al* (17) reported that good relationships between patients, families and medical personnel improved medication adherence. In the present study, similarly, confidence in the doctor or pharmacist affected medication adherence. However, if the guidance provided to the patient is insufficient and the patient does not understand the supportive care, it may lead to a decrease in the quality of life and an increase in the frequency of doctor visits and re-hospitalization due to improper or inadequate medication, including the use of antiemetics in supportive care (18).

Regarding the factors associated with treatment, the effect of the frequency, duration and number of drugs taken on medication adherence must be considered. It has been reported that an administration frequency of more than three times a day reduces medication adherence significantly (19). In the present study, the majority of the drugs were to be taken once or twice a day. Therefore, it was assumed that the effect of the medication administration frequency would be small. However, the patients with insufficient medication adherence felt that they take a large number of drugs, particularly when drugs were prescribed at the same time as the anticancer medication. Therefore, all drugs taken, not only anticancer agents, should be considered, as this could lead to an improved understanding of patient-specific issues and improve the directions offered to patients.

The attitude of a patient towards the disease and treatment is an important factor. In the current study, patients with good medication adherence were interested in learning about the medicine and disease, and actively participated in their treatment. It was also found that a positive attitude towards the disease could affect medication adherence. If patients express a positive attitude towards their disease, their willingness to participate in the monitoring of side-effects may be higher.

The health of the patients also affected medication adherence. In the present study, poor medication adherence was associated with poor patient health. It could be hypothesized that adherence to the treatment plan may be prevented by having poor health.

According to the CS analysis, the items requiring the greatest improvement were the interest in the drug, the desire to consult about the drug and the condition of the patient. The interest of patients in their own medication leads to the improvement of medication adherence, which has been demonstrated to alleviate anxiety; guiding these patients in the management of side-effects is necessary. In addition, interventions tailored to the condition of each patient are necessary to ensure that the proper medication regimen is fully adhered to. Winkeljohn (20) reported that health care providers should work with patients on intervention plans, including discussing anxiety or answering treatment questions, providing education about the symptoms of the disease and teaching the management of medication side-effects. Patient education and continuing guidance can be considered beneficial when delivered at appropriate times using appropriate methods.

In summary, medical personnel must be aware of the number of medications taken by the patient, the course of the medication and the age of the patient when they instruct patients undergoing anticancer treatments on the use of oral anticancer agents. Future studies are necessary for an improved understanding of the effect and necessity of drugs and their side-effects. In addition, interventions and instructions must begin to be tailored to the condition of each patient, as this would encourage patients to take a greater interest in their own medication. These actions may lead to improved medication adherence and enhance the efficacy, safety and continuity of oral anticancer treatments.

## Figures and Tables

**Table I:** Questionnaire.

**A questionnaire relating to taking your medication** Please answer the following questions by indicating the answers which apply to you (please circle the number or position which applies to you)					

Question	Yes	I think so	I cannot say either way	I do not think so	No
1. I understand how to take the medication	5	4	3	2	1
2. I know the effect (efficacy) of the medication	5	4	3	2	1
3. I know the side effects of the medication	5	4	3	2	1
4. I understand the current therapy	5	4	3	2	1
5. I agree with the current treatment policy	5	4	3	2	1
6. I have forgotten to take the medication or I have mistakenly taken the medication	Never	Rarely	I cannot say either way	Occasionally	Often
7. Currently, what is the composition of your household?	I live alone		Husband and wife		Other
8. I take care not to forget my medication	5	4	3	2	1
9. I think I take a lot of medications	5	4	3	2	1
10. Dosing times are complicated or awkward	5	4	3	2	1
11. The current treatment costs (prescription charges) are an economic burden	5	4	3	2	1
12. I believe the medication is necessary for me	5	4	3	2	1
13. I believe the medication is effective	5	4	3	2	1
14. I have actively researched about my medication	5	4	3	2	1
15. I worry about side effects	5	4	3	2	1
16. I would like to talk to someone further about the medication	5	4	3	2	1
17. I have a regular daily schedule	5	4	3	2	1
18. I eat regular meals	5	4	3	2	1
19. I am methodical	5	4	3	2	1
20. I am a worrier	5	4	3	2	1
21. I tend to find things bothersome	5	4	3	2	1
22. I have a good understanding of my disease	5	4	3	2	1
23. I trust the attending physician	5	4	3	2	1
24. I trust the pharmacist	5	4	3	2	1
25. I hope that the medication is valuable in curing the disease or that it will be able to improve my quality of life	5	4	3	2	1
26. I have a positive attitude towards the disease	5	4	3	2	1
27. What is your general condition (health)?	Very good	Good	Average	Bad	Very bad

Thank you for your cooperation					

**Table II:** Score of patient attribute factors that affect medication adherence.

	Medication adherence		
		
Factor	Good (n=109)	Insufficient (n=62)	P-value
Age, years^a^	66 (21–85)	73 (30–90)	0.0004
Gender, n			0.7249
Male	60	34	
Female	49	28	
Number of drugs taken (range)^a^,^b^	4 (1–10)	4 (1–14)	0.0401
Dosing time, days (range)^a^	131 (3–3585)	219 (24–3465)	0.0447
Stage, n			0.6609
Adjuvant	29	19	
Progressive	80	43	
Family, n			0.5692
Living alone	96	54	
Other	13	8	

a Statistically significant difference.

b Includes medication other than anticancer drugs.

**Table III:** Score of factors that affect medication adherence.

			Median medication adherence score (range)		
				
Topic	Question number	Question	Good (n=109)	Insufficient (n=62)	P-value
Living environment	7	Currently, what is the composition of your household?	2 (1–3)	3 (1–3)	0.9915
Awareness about dosing	8	I take care not to forget my medication	5 (4–5)	5 (3–5)	0.4968
	9	I think I take a lot of medications	2 (1–5)	3 (1–5)	0.0116^a^
	10	Dosing times are complicated or awkward	1 (1–5)	1 (1–5)	0.1819
Awareness about drug	11	The current treatment costs (prescription charges) are an economic burden	3 (1–5)	3 (1–5)	0.6207
	12	I believe the medication is necessary for me	5 (1–5)	5 (1–5)	0.1199
	13	I believe the medication is effective	5 (2–5)	5 (2–5)	0.0358^a^
	14	I have actively researched about my medication	2 (1–5)	1 (1–5)	0.0397^a^
	15	I worry about side effects	4 (1–5)	4 (1–5)	0.2185
	16	I would like to talk to someone further about the medication	3 (1–5)	2 (1–5)	0.0413^a^
Daily schedule	17	I have a regular daily schedule	5 (1–5)	5 (2–5)	0.1528
	18	I eat regular meals	5 (2–5)	5 (2–5)	0.3231
Personality	19	I am methodical	4 (1–5)	4 (1–5)	0.1716
	20	I am a worrier	4 (1–5)	3 (1–5)	0.1143
	21	I tend to find things bothersome	3 (1–5)	3 (1–5)	0.4135
Understanding of the stage of the disease	22	I have a good understanding of my disease	5 (2–5)	5 (1–5)	0.0262^a^
Sense of trust	23	I trust the attending physician	5 (3–5)	5 (1–5)	0.0262^a^
	24	I trust the pharmacist	5 (2–5)	5 (1–5)	0.0345^a^
Expectations and attitude	25	I hope that the medication is valuable in curing the disease or that it will be able to improve my quality of life	5 (2–5)	5 (2–5)	0.3717
	26	I have a positive attitude towards the disease	5 (3–5)	5 (1–5)	0.0245^a^
Condition	27	What is your general condition (health)?	3 (2–5)	3 (2–5)	0.0196^a^

a Statistically significant difference.

**Table IV:** Data obtained from CS analysis.

Question number	Topic	Average value of explanatory variables	Correlation coefficient	Average value of explanatory variables, deviation value	Correlation coefficient, deviation value	Angle	Distance	Degree of improvement
14	Interest in drug^a^	2.29	0.1544	35.28	50.83	41.77	14.743	11.00
16	Desire to consult about drug^a^	2.47	0.1367	37.09	49.12	48.91	12.937	8.50
27	Condition of patient^a^	3.45	0.1976	46.86	55.01	12.90	5.916	5.77
24	Confidence in pharmacist	4.44	0.2040	56.74	55.63	95.09	8.782	−0.78
22	Understanding of disease	4.58	0.2062	58.20	55.85	99.51	10.068	−1.66
23	Confidence in doctor	4.81	0.2241	60.42	57.58	98.97	12.884	−2.01
13	Drug effect	4.45	0.1455	56.85	49.97	135.29	6.853	−4.87
26	Positive attitude	4.78	0.1790	60.19	53.22	117.48	10.681	−4.93
9	Number of drugs taken	2.60	−0.1349	38.38	22.80	111.86	29.576	−11.01

a Items requiring improvement according to the customer satisfaction (CS) analysis.

## References

[1] Owashi T, Uejima K (2008). Adherence from compliance. Gekkan yakuji.

[2] Wu EQ, Guerin A, Yu AP (2010). Retrospective real-world comparison of medical visits, costs, and adherence between nilotinib and dasatinib in chronic myeloid leukaemia. Curr Med Res Opin.

[3] Reginster JY (2006). Adherence and persistence: impact on outcomes and health care resources. Bone.

[4] Macintosh PW, Pond GR, Pond BJ, Leung V, Siu LL (2007). A comparison of patient adherence and preference of packaging method for oral anticancer agents using conventional pill bottles versus daily pill boxes. Eur J Cancer Care (Engl).

[5] Marin D, Bazeos A, Mahon FX (2010). Adherence is the critical factor for achieving molecular responses in patients with chronic myeloid leukemia who achieve complete cytogenetic responses on imatinib. J Clin Oncol.

[6] Nilsson JL, Andersson K, Bergkvist A (2006). Refill adherence to repeat prescriptions of cancer drugs to ambulatory patients. Eur J Cancer Care (Engl).

[7] Noens L, van Lierde MA, De Bock R (2009). Prevalence, determinants, and outcomes of nonadherence to imatinib therapy in patients with chronic myeloid leukaemia: the ADAGIO study. Blood.

[8] Partridge AH, Archer L, Kornblith AB (2010). Adherence and persistence with oral adjuvant chemotherapy in older women with early-stage breast cancer in CALGB 49907: adherence companion study 60104. J Clin Oncol.

[9] Hershman DL, Kushi LH, Shao T (2010). Early discontinuation and nonadherence to adjuvant hormonal therapy in a cohort of 8,769 early-stage breast cancer patients. J Clin Oncol.

[10] Lebovits AH, Strain JJ, Schleifer SJ (1990). Patient noncompliance with self-administered chemotherapy. Cancer.

[11] Mayer EL, Partridge AH, Harris LN (2009). Tolerability of and adherence to combination oral therapy with gefitinib and capecitabine in metastatic breast cancer. Breast Cancer Res Treat.

[12] McCowan C, Shearer J, Donnan PT (2008). Cohort study examining tamoxifen adherence and its relationship to mortality in women with breast cancer. Br J Cancer.

[13] Moore S (2010). Nonadherence in patients with breast cancer receiving oral therapies. Clin J Oncol Nurs.

[14] Hasegawa K, Kuritani Y, Adachi A (2008). Improvement of drug compliance and pharmaceutical care - types of drug and dosing regimens desired by patients. Iryouyakugaku.

[15] Tsuboi K, Teramachi H, Kuzuya Y (2012). Survey of the patients’ consciousness affecting medication adherence. Iryouyakugaku.

[16] Ziller V, Kyvernitakis I, Knöll D (2013). Influence of a patient information program on adherence and persistence with an aromatase inhibitor in breast cancer treatment - the COMPAS study. BMC Cancer.

[17] Russmann S, Curkovic I, Huber M (2010). Adverse reactions and risks associated with non compliance. Ther Umsch.

[18] Ruddy K, Mayer E, Partridge A (2009). Patient adherence and persistence with oral anticancer treatment. CA Cancer J Clin.

[19] Lee CR, Nicholson PW, Souhami RL, Deshmukh AA (1992). Patient compliance with oral chemotherapy as assessed by a novel electronic technique. J Clin Oncol.

[20] Winkeljohn D (2010). Adherence to oral cancer therapies: nursing interventions. Clin J Oncol Nurs.

